# Role of prophylactic central compartment lymph node dissection in clinically N0 differentiated thyroid cancer patients: analysis of risk factors and review of modern trends

**DOI:** 10.1186/s12957-016-0879-4

**Published:** 2016-05-17

**Authors:** Giovanni Conzo, Ernesto Tartaglia, Nicola Avenia, Pier Giorgio Calò, Annamaria de Bellis, Katherine Esposito, Claudio Gambardella, Sergio Iorio, Daniela Pasquali, Luigi Santini, Maria Antonia Sinisi, Antonio Agostino Sinisi, Mario Testini, Andrea Polistena, Giuseppe Bellastella

**Affiliations:** Department of Anesthesiologic, Surgical and Emergency Sciences, Division of General and Oncologic Surgery, School of Medicine—Second University of Naples, Via Sergio Pansini 5, 80131 Naples, Italy; Endocrine Surgery Unit, University of Perugia, Perugia, Italy; Department of Medical, Surgical, Neurological, Metabolic and Geriatric Sciences, Second University of Naples, Naples, Italy; Department of Surgical Sciences, University of Cagliari, Cagliari, Italy; Department of Cardio-Thoracic and Respiratory Sciences, Endocrinology Unit, Second University of Naples, Naples, Italy; Department of Biomedical Sciences and Human Oncology, Unit of Endocrine, Digestive and Emergency Surgery, University Medical School “A. Moro” of Bari, Bari, Italy

**Keywords:** Total thyroidectomy, Papillary thyroid cancer, Routine central lymph node dissection, Lymph node neck dissection, Radioactive iodine ablation

## Abstract

In the last years, especially thanks to a large diffusion of ultrasound-guided FNBs, a surprising increased incidence of differentiated thyroid cancer (DTC), “small” tumors and microcarcinomas have been reported in the international series. This led endocrinologists and surgeons to search for “tailored” and “less aggressive” therapeutic protocols avoiding risky morbidity and useless “overtreatment”. Considering the most recent guidelines of referral endocrine societies, we analyzed the role of routine or so-called prophylactic central compartment lymph node dissection (RCLD), also considering its benefits and risks. Literature data showed that the debate is still open and the surgeons are divided between proponents and opponents of its use. Even if lymph node metastases are commonly observed, and in up to 90 % of DTC cases micrometastases are reported, the impact of lymphatic involvement on long-term survival is subject to intensive research and the best indications of lymph node dissection are still controversial. Identification of prognostic factors for central compartment metastases could assist surgeons in determining whether to perform RLCD. Considering available evidence, a general agreement to definitely reserve RCLD to “high-risk” cases was observed. More clinical researches, in order to identify risk factors of meaningful predictive power and prospective long-term randomized trials, should be useful to validate this selective approach.

## Background

Differentiated thyroid cancer (DTC) is a relatively uncommon malignancy representing 1–2 % of all human malignancies with a worldwide mean annual incidence per 100,000 individuals ranging from 1.2 to 2.6 in men and from 2.0 to 3.8 in women, with a surprising increase in the last decades [1–5]. Papillary thyroid cancer (PTC) is the most frequent variant, and recently in the USA, its incidence is increased more than 240 % with 62.980 expected new cases in 2014. Thanks to US-guided FNBs, more and more tumors less than 2 cm and microcarcinomas were diagnosed [6–12]. According to main international series, better oncological outcomes are expected in these cases, and consequently, a less “aggressive” and “tailored” multimodal approach has been adopted to avoid a useless “overtreatment” and risky morbidity. Finally, different scientific societies suggested an accurate evaluation of laboratoristic, instrumental, clinical and genetic risk factors before to intimate a more “aggressive” therapeutic approach [13, 14]. Considering the high rate of lymph node metastases routine or so-called prophylactic central compartment lymph node dissection (RCLD) in clinically N0 patients is a matter of intensive research and is still debated [15, 16]. Endocrine surgeons, head and neck surgeons and otolaryngologists are divided between supporters and detractors of its use. In the last decade, in order to reduce locoregional relapse (LR) rate and thyroglobulin (Tg) serum levels, a trend toward routine dissection, avoiding radioactive iodine (RAI), has been generally reported. Nevertheless, lastly, considering evidence-based medicine (EBM) data, several authors suggested its avoidance in clinical practice reserving prophylactic dissection in high-risk patients [17–20]. The absence of macroscopic lymph node metastases node dissection might determine an overstaging of disease and a risky overuse of RAI that is not associated to better oncological outcomes in terms of LR and long-term survival in every case. Moreover, a not negligible morbidity should be taken into account.

So, in order to avoid risky “overtreatment” in low-risk patients or at the same time underestimate the real oncological status, identification of pre- and perioperative risk factors of meaningful predictive power should be considered subject to intensive research and of paramount interest.

Performing a literature review and considering the available evidence, in an attempt to better clarify the suitable indication and extension of routine central compartment neck dissection, we reviewed indications to lymphatic dissection in the current management of DTCs. Clinical experience, inhering the management of thyroid cancer in the last two decades and of the endocrine and endocrine surgery centers participating in the study, were also taken into account.

## Review

### Study design

LD, lateral or central LD, modified radical neck dissection, RCLD, selective, prophylactic or therapeutic LD, DTC and PTC were used as key words to perform a PubMed database research. The most recent guidelines regarding neck dissection for PTC according to the American Thyroid Association (ATA), European Thyroid Association (ETA), Unità operative di Endocrinochirurgia (UEC), American Head and Neck Society and American Academy of Otolaryngology-Head and Neck Surgery, Japanese Society of Thyroid Surgeons and Japanese Association of Endocrine Surgeons (JSTSJAES) and European Society of Endocrine Surgeons (ESES) were also considered. In particular, regarding terminology of cervical lymphatic anatomy (neck levels) and classification of neck dissection, the most recent ATA guidelines were considered [8]. LD benefits and risks, available evidence, complications and impact on locoregional recurrence rate and mortality were also evaluated.

### Risk factors

Researchers try to identify thyroid cancer predictive risk factors, pre-existing concauses of cancer onset or cancer-associated conditions that should be considered especially in preoperative evaluation of uncertain neoplastic lesions (Table 1). The most important environmental and exogenous factors are X-rays and ^131^I exposure, iodine and endocrine disruptors, while the endogenous ones are gender and age, TSH, autoimmunity, obesity and insulin resistance, hereditary conditions and family history.

**Table 1:** Risk factors for thyroid cancer

Esogenous	Endogenous
X-ray and ^131^I exposure [21–25]	Gender (*male*) and age (*<15 or >45 years*) [39–43]
Endocrine disruptors	TSH [23]
	Autoimmunity [22,24]
	Obesity and insulin resistance [29–33]
	Family history of thyroid cancer (*5 %*), hereditary conditions (*familial non-medullary carcinoma, familial adenomatous polyposis,* etc.) [26–28]

Exposure to head or neck radiation in childhood is a proven risk factor correlated to the intensity of radiation and the age of the child, increasing with larger doses and with younger age at treatment [21]. Moreover, X-ray and ^131^I exposure may increase thyroid risk cancer also in adult as well as autoimmune thyroiditis or prolonged iodine deficiency, associated to elevated TSH serum levels [22–25].

The gender disparity in incidence, aggressiveness and prognosis of thyroid cancer is well established. A possible role in the sex-related different biologic behavior might be played by the difference in the estrogen receptor subtypes expressed in tissue but the argument is still subject to controversies and the substantial causes have to be still clarified [26]. A family history of thyroid cancer is present in about 5 % of the patients and interesting researches have been reported. Usually, the familial non-medullary carcinoma is mostly of papillary histotype more aggressive than the sporadic forms with an incidence of 6.2–10.5 % and an autosomal, polygenic, dominant transmission but with incomplete penetrance [1]. PTC may furthermore occur in patients with familial adenomatous polyposis and its subtype Gardner’s syndrome, both sharing etiopathogenic defects in the gene APC [27, 28]. A high risk of papillary or follicular thyroid carcinoma has been also described in patients with Cowden’s disease and in people affected by Carney complex type I [1].

A higher BMI and incorrect eating habits, such as the excessive use of butter, cheese, starches and smoked fish, are moreover associated to an increased risk while a diet rich in fruits and vegetables seems to play a protective role [29–32]. Moreover, recent studies suggest the possibility that insulin resistance and hyperinsulinemia, a typical feature of obesity and metabolic syndrome, may be a risk factor for thyroid cancer [5, 33].

The role of some environmental pollutants as endocrine disruptors interfering with hypothalamic-pituitary-thyroid axis secretions is subject to intensive research and definitive conclusions were not reported [5, 34]. Finally, an interesting paper demonstrated an increased incidence of papillary thyroid microcarcinoma in Sicily, especially in the volcanic area, where it was more aggressive in young patients [35].

### Prognostic factors: state of art

There is no consensus regarding RCLD in PTC patients, and identification of prognostic factors for central lymph node metastases (CLNM) could assist surgeons in determining whether this procedure should be performed. Therefore, pre- and intraoperative risk factors for level VI metastases are of paramount interest and subject to intensive research.

Several papers yielded conflicting results due to variations in the study settings and in the observed population [36–38].

Factors increasing the risk of CLNM include the following: tumor size >1 cm, aggressive variants of PTC, extra thyroidal extension, tumor multifocality, age >45 or <15 years, male gender, white race, familiality, BRAF V600 mutation [39–41]. Nevertheless, literature results are not conclusive and still matter of debate. As well, recognized age has been undoubtedly reported to be a risk factor. The cut-off of 45 years is widely used as a clinical marker for prognosis [42]. In fact traditionally, patients older than 45 years are more often associated with poor prognosis and increased recurrence, as well as frequently reported for child <15 years [43].

A recent meta-analysis has observed that age younger than 45 years is a significant risk factor for CLNM in CN0 patients [44].

Although the incidence of thyroid cancer is higher in women, the rates of malignancy and mortality due to thyroid cancer are higher in men [45]. So the male sex can be considered as a risk factor [44].

Tumor size is another important factor in TNM staging, and large tumors are more prone to be aggressive [46]. The tumor size has been repeatedly confirmed as an independent predictor of both pathologic and clinical outcomes. Lymph node metastasis is known to increase with tumor size, and moreover, Jeong et al. showed an association between large neoplasms and LN metastases [47]. In their study, mean tumor size was greater in N+ cases compared to N0 patients (1.59 ± 1.03 vs 0.93 ± 0.62 cm; *p* < 0.001). Literature meta-analysis confirmed that larger tumors (>1 cm) were associated with an increased risk of CLNM [44].

Lim et al., in a previous study, had reported that tumor size (>5 mm) was a significant predictive factor of CLNM in PTC microcarcinoma [48]. Machens et al. also had demonstrated that PTC microcarcinoma of >5 mm were more associated with poor prognostic factors compared with those of <5 mm [49].

BRAF mutations have been found in various cancers including melanoma, colon cancer, and thyroid cancer, and in PTCs BRAFV600E mutation, a T1799A point mutation in the B-type Raf kinase gene is thought to be the most common genetic alteration related to tumor aggressiveness and poor prognosis [50–55].

Moreover, BRAFV600E mutation was independently related to known unfavorable prognostic factors such as extrathyroidal invasion, lymph node metastases, advanced tumor stage (III/IV) and aggressive subtypes. In fact, it was associated to PTC recurrence, even in low-risk groups [53]. Finally, in a retrospective multicenter study, BRAFV600E mutation-positive patients experienced more deaths per 1000 person-years than their wild-type counterparts (11.80 vs 2.25, hazards ratio 1⁄4 3.53) [54].

A recent meta-analysis, including 20 studies and 9084 patients that had undergone thyroidectomy + prophylactic central lymph node dissection (PCLND), extensively focused on the risk factors for central lymph node metastasis (CLNM) in patients with clinically negative central compartment lymph nodes [44]. As results, the following variables were associated with an increased risk: age <45 years, male sex, multifocality, tumor size >2 cm for PTC and >0.5 cm for papillary microcarcinoma, location of primary tumor in the central area and low lobe, lymphovascular invasion, capsular invasion and extrathyroidal extension. Instead, bilateral tumors and lymphocytic thyroiditis did not show association with increased risk of CLNM in these patients. The authors concluded that these factors should guide the application of PCLND in patients with clinically negative central compartment lymph nodes.

In conclusion, identification of PTC clinicopathological risk factors is crucial to improve the accuracy of recurrence rate estimates and to facilitate the calculation of patient‐specific disease mortality rates. Furthermore, it could allow a better selection of therapeutic protocol facilitating modality of follow-up [56].

### Lymph node dissection: definition and rationale of modern trends

CLNM are very frequent while lateral ones are rare and might be associated to a worse prognosis. In most cases, contralateral central or lateral spreading follows ipsilateral metastases, but “skip lesions” may be observed in about 10 % of patients, especially in superior pole cancers. In clinically N0 patients, the most suitable dissection is still debated because of uncertain prognostic value of micro and macro nodal metastases on oncological outcomes. The obscure significance of node involvement is the main cause of this unsolved issue. Thyroid cancer represents a unique and atypical neoplasm mostly associated to a favorable prognosis. Dissimilarly to all other cancers originating from different anatomic districts—chest, gastrointestinal, reproductive system….—in which unavoidably lymph node metastases are associated to a worse prognosis, in DTC they are not synonymous of more aggressive biological behavior and are not associated to unfavorable outcomes. Nevertheless, in clinically N+ patients, a higher rate of persistent or recurrent disease is mostly reported, and in “high-risk” cases, node metastases might affect long-term survival [16, 57]. According to Smith et al., who reported an analysis of about 11,000 cases, in clinically N+ patients >45 years lateral nodes are associated to a worse prognosis respect to younger patients with central compartment metastases [58].

Moreover, the surprisingly elevated incidence of microscopically positive lymph nodes, their natural evolution and their not frequent progression to a clinical recurrence represent the second obscure phenomena that should be clarified by more and more researches. All therapeutic efforts in order to eradicate microscopic disease do not favorably modify just fair oncological outcomes placing patients to risk of useless “overtreatment” with long-term unfavorable side effects.

In clinical management of node metastases, several oncological principles are well-known dogmas while other ones are still debated. First of all, an accurate staging is recommended but physical examination and cervical ultrasound are still critical in the preoperative work-up because in about one third of cases unexpected node metastases are successively discovered. So an accurate intraoperative inspection from hyoid bone to sternal notch by an expert surgeon is mandatory to avoid missing residual disease postoperatively associated to higher Tg serum levels and recurrence rate [59]. Explicit and clear communication between specialists about prior operations (extent of disease and sublevels of dissection) is very important to avoid risky interventions and facility surgical management (scarred surgical bed). Until the anatomic node classification and definition of neck dissection by American Society of Head and Neck Surgery and successively by ATA [8], operative reports were unable to accurately describe lymphatic involvement and extension of dissection performed. Consequently, retrospective analysis of surgical results was unreliable and outcomes incomparable. Thanks to specific anatomic landmarks, nodes were accurately divided into cervical and mediastinal levels (I–VII) and moreover grouped into central (I, VI and VII) and lateral neck compartment (II–V). Central and lateral neck dissections were described by a published consensus statement on the terminology and classification [60, 61].

So neck dissection is nowadays performed by a standardized and widely diffuse surgical approach. Selective lymph node dissection (central LD is its variant) introduced by Ballantyne in 1980—consisting en bloc removal of all lymphatic fibrous adipose tissue along with specific fascial planes—or modified radical neck dissection (MRND) firstly described by Suarez-Bocca in 1967—en block removal of all neck levels (I–VII) with respect of jugular vein, sternocleidomastoid muscle and accessory spinal nerve—became the operations of choice in DTC treatment. Radical neck dissection, associated to higher morbidity and “berry picking” followed by higher recurrence rate, are mostly contraindicated.

In case of clinical node involvement, a compartment-oriented resection of the entire lymphatic basin—systematic selective ipsilateral or bilateral, central or lateral dissection, or mono or bilateral MRND—is recommended according to risk categories in order to obtain a lower recurrence rate and a higher survival [62, 63]. In clinically N+ “high-risk” patients, in the presence of more than five metastatic lymph nodes or of one node greater than 3 cm in diameter, a selective lateral dissection may be associated to a central compartment one (levels III–IV) [64]. In patients affected by lateral metastases, central and lateral neck dissection is required (levels II, III, IV, VI), reserving a bilateral dissection, in case of multiple metastases, considering the elevated incidence of contralateral central neck metastases demonstrated in the surgical specimens.

Conversely, in the absence of involved nodes, the role of prophylactic LD is still debated [65, 66]. According to its proponents, RCLD, defined as complete excision of the levels VI and VII (considering the recognized anatomical continuity from neck and superior mediastinum) might be safely performed avoiding to miss virulent disease, allowing a better chance of cure with a low morbidity, and reducing postoperative Tg serum levels and recurrence risk. The following should be suggested: considering higher morbidity of reoperations, removing potential source of recurrence, improving diagnostic accuracy, simplifying the follow-up and finally modifying the indications to RAI [67–69]. Caliskan et al. suggested that the central compartment dissection is technically feasible and safe representing the best way to determine node status for a more accurate staging and risk stratification [69]. Nevertheless, it is generally associated with a higher rate of transitory complications and according to Barczynski et al. and T.S. Wang et al., it is contraindicated in low-volume centers [70, 71]. Higher morbidity rate, the uncertain clinical significance of node involvement, absence of proven benefits on survival, a consequent up staging and finally a RAI overuse with undesirable side effects such as nausea, vomiting, ageusia, salivary gland swelling, sialoadenitis, xerostomia, pulmonary fibrosis, dental caries and second primary malignancies (0.5 %) are advocated against routine LD. Moreover, a similar risk of local recurrence—0–9 %—was reported in clinically N0 patients who undergone RCLD or TT alone [8], differently from N+ cases (relapse rate up to 40 %).

The most common questions are as follows: Does RCLD reduce locoregional recurrence? Does it increase morbidity? Does it increase the morbidity in patients that have to be re-operated on the central compartment? According to interesting meta-analysis and evidence-based medicine (EBM) studies, RCLD might reduce locoregional recurrence (level IV–V, no recommendation), improve disease-free survival (grade C), increase the number of patients with undetectable levels of Tg (level IV, no recommendation) and increase permanent hypoparathyroidism and recurrent nerve lesions (grade C). It must be performed by experienced hands (C) [67, 72]. Finally, central compartment reoperations increase permanent hypoparathyroidism and recurrent lesions. Recently, Barczynski et al. stated that RCLD upstages PTC patients determining risky overuse of RAI that is not associated to a better outcome [70]. Nevertheless, several authors demonstrated that most LN recurrences are in the lateral compartment (levels III–IV) reducing the supposed RCLD benefits [73].

Moreover, micrometastases do not affect clinical course and outcome of PTC patients [20]. *In conclusion*, routine LD allows a better staging and RAI selection reducing in some cases Tg serum level and recurrence rate but nevertheless is associated to higher risk of complications. According to literature data, Table 2 reports proponents and opponents of prophylactic node dissection testifying that the debate is still open.

**Table 2:** Proponents and opponents of RCLD

	Pros			Cons	
Y Ito et al.	WJS	2006	DL Steward et al.	Thyroid	2019
CL Lundgren et al.	Cancer	2006	WT Shen et al.	Surgery	2010
M Shindo et al.	Arch OHN J	2006	AR Shaha et al.	Cure Op Ot HNS	2011
M Sywak et al.	Surgery	2006	MA Moreno et al.	Thyroid	2012
ML White et al.	WJS	2007	DE Gyorki et al.	Ann Surf Oncol	2013
JL Roh et al.	Ann Surg Onc	2008	G Conzo et al.	Endocrine	2013
W Hu et al.	AI Zheng	2008	K Zanocco et al.	Surgery	2013
YI Son et al.	Ann Surg Onc	2008	L Santini et al.	Surgery	2014
N Palestini et al.	Arch. Surg	2008	PG Calò et al.	WJSO	2014
S Bonnet et al.	ICEM	2009	P Miccoli et al.	JCEM	2015
Y Giles et al.	Surgery	2009			
G Senyurek et al.	Surgery	2009			
BM Sadowski et al.	Surgery	2009			
YK So et al.	Surgery	2010			
TA Moo et al.	WJS	2010			
Sui-Zhou Xiao et al.	WJS	2010			
P Caglia et al.	G. Chir.	2010			
YS Lee et al.	WJS	2010			
M Barczyński et al.	Br J Surg	2013			
TS Wang et al.	Ann Surf Onco	2013			
Q Wang et al.	Clin Transl Oncol	2014			
P Summan et al.	Surgery	2015			

In addition, analyzing the most recent series, a similar recurrence rate was reported in patients undergone TT alone or associated to routine LD, reducing presumed advantages of prophylactic operations (Table 3).

**Table 3:** Relapse incidence following TT with or without RLCD (%)

Author	Journal/year	TT	TT with RLCD
M Sywak et al.	Surgery 2006	5.6	3.6
S Costa et al.	Acta Oto Ita 2009	6.8	7.1
DT Hughes et al.	Surgery 2010	4.6	5.1
TA Moo et al.	WJS 2010	16.7	4.4
WT Shen et al.	Surgery 2010	5.7	21.8
M Raffaelli et al.	Surgery 2012	0.0	1.6
BH Lang et al.	Ann Surg Oncol 2012	2.9	3.7
M Barczyński et al.	Br J Surg 2013	13.1	4.2
M Barczyński et al.	Lange Arch Surg 2014	–	6.3
DM Hartl et al.	WJS 2013	12	2
G Conzo et al.	Surgery 2014	3.8	3.3
DE Gyorki et al.	Ann Surg Oncol 2013	2.2	1.8

The adoption of risk factors in stratifying patient categories remains of paramount importance to avoid useless RCLD. As reported above, age <45 years, multifocality, familiality, male sex, aggressive pathological variants, BRAF V600 mutation and tumor size larger than 1 cm are the main independent preoperative variables. They should be considered in association with extra capsular thyroid infiltration, positive margins, and lymphovascular invasion that might be intra- or postoperatively acquired.

Therefore, different selecting criteria were suggested to identify the best RLCD indications. Several surgeons are in favor of its use in high-risk cases, in presence of positive frozen section or biopsy-proven disease and in lateral clinically node-positive patients. In addition, ipsilateral routine dissection plus frozen section (and eventually contralateral dissection) was recently introduced considering a high morbidity rate of bilateral procedure and that isolated contralateral LN metastases are exceptionally described. This tailored approach showed similar staging ability to that observed after bilateral RCLD and a lower morbidity rate similar to that reported after TT alone. The main limit consisted in overlooking contralateral metastases with a higher recurrence rate [74].

In the absence of adequate statistical power to demonstrate clear benefits on long-term outcomes, more prospective clinical trials are needed.

The most recent ATA and UEC guidelines stated that prophylactic LD could be considered in high-risk patients with advanced primary tumors and should be performed by high-volume surgeons to avoid definitive complications [62]. A reduced local recurrence rate and a lower Tg serum level may be expected. The procedure allows a better staging too [68], but a prospective randomized study on RCLD role could be very expensive and not readily feasible [75]. Revisiting the 2014 Japanese Society of Thyroid Surgeons and Japanese Association of Endocrine Surgeons (JSTSJAES) guidelines, it has been stated that in the absence of definitive data about prophylactic CND in a large series of patients, its indication depends on institutional policy and surgeons’ skill levels [76]. On the contrary, Consensus European Society of Endocrine Surgeons (ESES) confirmed that routine level VI prophylactic dissection should be risk stratified in T3–T4 cases, in patients >45 or <15 years, male patient, bilateral or multifocal tumors, lateral known involved lymph nodes [77].

In our experience, a clinical retrospective study on 221 cases, TT followed by RAI administration and TSH suppression therapy, guaranteed optimal long-term results, with a low incidence of locoregional recurrence similar to that reported in patients undergone TT alone [16]. Reoperations were usually not associated with higher morbidity, especially performing unilateral dissection, although hypoparathyroidism and unintentional recurrent laryngeal nerve injury have been observed in up to 14 and 9 % of patients, respectively [78].

In the absence of enlarged lymph node, and when RAI administration is advisable (tumor >2 cm in a male patient >50-year-old), routine lymph node dissection might be not indicated, while, in low-risk patients with tumors ≤1 cm, RCLD may discover metastases requiring RAI ablation, modifying the therapeutic protocol [68]. Nevertheless, in these patients, the RAI advantages remain to be proven. Gyorki et al., in favor of therapeutic central neck dissection, in a recent assessment of clinical evidence, hypothesized that node positivity, following prophylactic dissection, may encourage administration of higher doses of ^131^I without obvious benefit [79].

Finally, analyzing potential oncological benefits and morbidity rate, routine dissection of level V (a/b) is still a controversial topic, reserving its indication only to N+ cases [80].

## Conclusions

In the treatment of DTC, considering a surprising increasing rate of a precocious diagnosis of tumors less than 2 cm and of microcarcinomas and the better oncological outcomes expected, a “tailored” and “less aggressive” multimodal therapeutic approach should be suggested, to avoid unfavorable even if minimal morbidity following a potential “overtreatment”.

In the absence of involved lymph nodes, prophylactic dissection should be avoided, reserving RCLD to “high-risk” patients to reduce the local recurrence rate. More researches are needed in order to identify pre- and perioperative risk factors of predictive power useful in planning tailored therapeutic protocols.
